# Bullying behaviour in schools, socioeconomic position and psychiatric morbidity: a cross-sectional study in late adolescents in Greece

**DOI:** 10.1186/1753-2000-6-8

**Published:** 2012-02-12

**Authors:** Konstantina Magklara, Petros Skapinakis, Tatiana Gkatsa, Stefanos Bellos, Ricardo Araya, Stylianos Stylianidis, Venetsanos Mavreas

**Affiliations:** 1 Department of Psychiatry, University of Ioannina, School of Medicine, Ioannina, Greece; 2 Academic Unit of Psychiatry, School of Social and Community Medicine, University of Bristol, Bristol, UK; 3 Department of Psychology, Panteion University of Social and Political Sciences, Athens, Greece; 4 Department of Psychiatry, University of Ioannina, School of Medicine, Ioannina 45110, Greece

**Keywords:** Bullying, Adolescents, Socioeconomic status, Psychiatric morbidity, Victims, Perpetrators

## Abstract

**Background:**

Bullying is quite prevalent in the school setting and has been associated with the socioeconomic position and psychiatric morbidity of the pupils. The aim of the study was to investigate the association between bullying and socioeconomic status in a sample of Greek adolescents and to examine whether this is confounded by the presence of psychiatric morbidity, including sub-threshold forms of illness.

**Methods:**

5,614 adolescents aged 16-18 years old and attending 25 senior high schools were screened and a stratified random sample of 2,427 were selected for a detailed interview. Psychiatric morbidity was assessed with a fully structured psychiatric interview, the revised Clinical Interview Schedule (CIS-R), while bullying was assessed with the revised Olweus bully/victim questionnaire. The following socio-economic variables were assessed: parental educational level and employment status, financial difficulties of the family and adolescents' school performance. The associations were investigated using multinomial logit models.

**Results:**

26.4% of the pupils were involved in bullying-related behaviours at least once monthly either as victims, perpetrators or both, while more frequent involvement (at least once weekly) was reported by 4.1%. Psychiatric morbidity was associated with all types of bullying-related behaviours. No socioeconomic associations were reported for victimization. A lower school performance and unemployment of the father were significantly more likely among perpetrators, while economic inactivity of the mother was more likely in pupils who were both victims and perpetrators. These results were largely confirmed when we focused on high frequency behaviours only. In addition, being overweight increased the risk of frequent victimization.

**Conclusions:**

The prevalence of bullying among Greek pupils is substantial. Perpetration was associated with some dimensions of adolescents' socioeconomic status, while victimization showed no socioeconomic associations. Our findings may add to the understanding of possible risk factors for bullying behaviours in adolescence.

## Background

Bullying is quite prevalent in the school setting and has important adverse effects on many areas of the adolescents' life. It is a specific type of aggression in which an intension to harm or disturb can be identified, occurs repeatedly over time and there is an imbalance of power, with a more powerful person or group attacking a less powerful one [1]. According to a widely used research definition of bullying a student is being bullied or victimized when he or she is exposed, repeatedly and over time, to negative actions on the part of one or more other students [2]. Negative actions are further defined as when someone (the "perpetrator") intentionally inflicts, or attempts to inflict, injury or discomfort upon another (the "victim"). Negative actions can be verbal, including threatening, taunting, teasing, or name-calling, or physical, such as hitting, kicking, pushing, shoving or pinching. Being a victim of bullying has been associated with lower self-esteem [3], depressive symptomatology [4-7], anxiety [3], physical and psychosomatic symptoms [8-10], suicidal ideation [11,12] and suicide [13]. On the other hand, being a perpetrator has been associated with aggression [14], antisocial personality, criminality and substance abuse [15]. As regards the direction of causality, studies have shown that bullying appears to be a potential risk factor for mental health problems, since it usually precedes the onset of emotional difficulties [5].

A large number of studies conducted in different countries indicate that bullying at school occurs all over the world and is not confined to any geographical region, socioeconomic or cultural group. The prevalence, however, of bullying varies considerably between countries. Studies show prevalence rates of the overall phenomenon between 8% in Germany [16] and 29.9% in the United States [17], 30% in Italy [18] and 40% in Korea [19]. Prevalence rates of perpetrators vary between 4% and 50% [20], while rates of victims of bullying vary between 4.1% for girls in Sweden and 36.3% for boys in Lithuania [21]. A recent international study which investigated the prevalence of bullying victimization in 66 countries and territories reports that on average 32,1% of the children were bullied at school at least once within the past 2 months, while 37,4% of children were bullied at least one day within the past 30 days [22]. Boys are more often perpetrators than girls [23], while rates of victimization may not differ between the two genders [17,24,25]. Both behaviors appear to be more common in younger ages [17,26,27].

According to Craig et al. the prevalence of bullying combined (i.e., bullying others, being bullied and being both a bully and a victim) in Greece was 41.3% and Greece occupied the third place among 40 countries in the number of adolescent students being involved in bullying-related behaviours [23]. Possible explanations of this relative high prevalence are the lack of national policies against bullying in Greece, as well as a number of cultural variations, for example the way bullying is conceptualized and understood [28].

The cross-national variations in the prevalence of bullying may reflect the different distribution of culture-specific risk factors among countries and the different methods used in research. Regarding socioeconomic status, lower parental education [26] and poorer academic achievement of the student in school have been associated with bullying [17]. Recently, an international study showed that being a victim was more common among adolescents from families of lower socioeconomic position and this association appeared to be relatively strong across several countries [29]. Another study, which investigated socioeconomic associations of bullying using a sample of preschool children, has also shown that children from families with lower educational level present an increased risk of victimization [30] A study conducted in Germany and England has also reported associations between social class and both victims and perpetrators [16]. Moreover, not only the presence but also the persistence of bullying over time has been associated with lower socioeconomic status of the family [31]. A recent review suggests that bullying is not only a socially patterned life experience, but it tracks over time and there are indications of a socially differential vulnerability to its effects. Exposure to bullying may be an element of a pathway through which socioeconomic position in adolescence contributes to adult health inequalities [32]. At the school level, Whitney and Smith (1993) reported that junior and middle schools with higher proportions of families from lower social classes had a higher prevalence of bullying [24]. Finally, at the country level countries with higher income inequality had a higher prevalence of bullying among preadolescents than countries with lower income inequality [33].

As described in the previous paragraphs psychiatric morbidity has been associated with bullying-related behaviours. In addition previous studies have shown a strong association between several socioeconomic variables and psychiatric morbidity [34-36]. Therefore, an important confounding variable in the association between socioeconomic status and bullying is the presence of psychiatric morbidity. Not all previous studies have adjusted for the full spectrum of psychiatric morbidity including sub-threshold forms of illness. It is likely that a more detailed assessment of psychiatric morbidity including also sub-threshold forms of illness could explain part of the confounding and could reduce possible associations between bullying and socioeconomic status. It is noted that confounding is an important issue irrespective of the study design and could influence the results of both cross-sectional and longitudinal studies. Clarifying whether low socioeconomic status is associated with bullying after adjustment for all potential confounding factors may contribute to the discussion about possible causal pathways of bullying-related behaviours.

The aim of the present study was to investigate the association between bullying-related behaviours (being either a victim or a perpetrator or both) and socioeconomic status in a sample of adolescents attending senior high schools in Greece. Since the burden of psychiatric morbidity is significant in adolescence [37], we explored possible effects of the full spectrum of psychiatric morbidity on the association between bullying and socioeconomic indicators. We made the hypothesis that socioeconomic indicators would be independently associated with bullying-related behaviours after adjusting for psychiatric morbidity. We have also investigated the association of obesity problems with bullying-related behaviours, since there is evidence that obesity or overweight may be associated with both bullying and socioeconomic status [38-40].

## Methods

### Description of the data set

The data reported here are derived from the "Epirus School Project" [35] which is a cross-sectional survey carried out in selected upper secondary schools in Greece. The study was approved by the Ethical committee of the Ministry of Education and the Greek Pedagogical Institute (decision number: 61390/g2/19-06-2006) and was conducted according to the Helsinki declaration. The study was also approved by the Head of each participating School. The principal aim of the survey was to investigate the prevalence and associations of common mental disorders in late adolescence.

### Secondary education in Greece

Secondary education in Greece is distinguished into lower secondary (grades 7-9; ages 13-15 years; attendance is compulsory) and upper secondary (grades 10-12; ages 16-18 years; attendance is not compulsory). Upper secondary schools are further distinguished into senior high schools (Lyceum) and technical vocational schools with the majority of students (75%) attending senior high schools. In the "Epirus School Project" only senior high schools were selected (age of pupils 16-18 years). At the time of the design of the study approximately 75,000 students attended 1,193 senior high schools.

### Sampling of schools and pupils

Schools were selected according to the following rules: a) all senior high schools of the major cities in the north-western part of Greece (regions of Epirus and Aetoloakarnania) due to the proximity of this area to the University of Ioannina, b) all senior high schools in one randomly selected district of the Athens Metropolitan Area (the district of Kallithea was selected), c) all senior high schools of the island of Paros in the Aegean Sea (the island was conveniently selected). A total of 25 schools took part in the study. The median number of participants per school was 225 pupils ranging from 138 to 425. The main fieldwork took place between January 2007 and April 2008. All students in the selected schools were invited to participate in the study, while the participation was voluntary. Consent was actively obtained from both the participants and their parents.

### Design of the study and data collection procedure

The study used a two-phase design [41]. In the first phase, all consenting students (N = 5,614, response rate 82%) were administered a brief screening instrument in the classroom. The screening instrument of the first phase was developed from the revised clinical interview schedule (CIS-R) used in the second phase of the study. Students were selected for the second phase psychiatric interview using a stratified random sampling procedure according to the scores on the screening questionnaire: 100% of those scoring high on the screening instrument (> 75th percentile), 30% of those scoring in the middle and 10% of those scoring low (< 25th percentile). The second phase (N = 2,431, response rate 95%) consisted of the computerized version of a fully-structured psychiatric interview (see next section) and was carried out in the computer laboratories of the schools. It is noted that in two schools (both in the island of Paros) all consenting students were interviewed (that is the two phases were merged into one). The reason was the availability of the fieldworkers of the island of Paros, which allowed us to provide the instrument of the CIS-R interview in full to all consenting students. From the remaining 1,960 pupils who were selected according to the stratified random sampling procedure, 926 (47.2%) were on the 100% stratum, 866 (44.2%) on the 30% stratum and 168 (8.6%) on the 10% stratum. Four out of the 2,431 selected pupils had missing values on the sociodemographic questions (administered in the first phase of the study) and therefore 2,427 pupils were used in the final analysis.

### Assessment of psychiatric morbidity: the revised clinical interview schedule (CIS-R)

Psychiatric symptoms were assessed with the revised clinical interview schedule (CIS-R), a fully structured psychiatric interview designed to be used by trained lay interviewers [42]. The CIS-R was the main instrument used in the national psychiatric morbidity surveys in the UK [43] and has been used in several other similar surveys around the world. A computerized version has also been developed and found to be comparable with the regular interview [44]. The CIS-R was originally designed to assess symptoms in participants above 16 years old but has been previously used in teenagers above 14 years old in Australia [45]. The CIS-R assesses the presence and severity of common psychological symptoms (somatic symptoms, fatigue, concentration/memory problems, sleep problems, irritability, depressive mood, depressive ideas, general worry, worry about physical health, free-floating anxiety, phobias, panic anxiety, compulsions and obsessions). Two screening questions in each section ask about the presence of the symptom during the past month and then there is a more detailed assessment of the presence, frequency, duration and severity of the symptom during the past seven days. Based on the above-mentioned characteristics of the symptoms each one of the 14 symptoms is rated with an individual score on a scale ranging from 0 to 4 (except depressive ideas scored from 0 to 5).

In the first phase of the study we used the screening questions of the several symptom sections of the CIS-R. To simplify the screening we excluded the somatic symptoms section, the depressive ideas section (since it is branched through the depression section), and the panic section (it is branched through the anxiety section). We used the main screening question for the symptoms of fatigue, concentration problems, memory problems, worry about physical health, free-floating anxiety, phobias, obsessions, and compulsions (eight questions) and the two screening questions for the symptoms of sleep problems (one question for difficulty sleeping and one for sleeping more than usual), irritability, depression (depressed mood and loss of interest) and anxiety (eight questions). Therefore the screening instrument consisted of 16 yes-no questions. The full interview was taken by those students selected for the second phase of the study.

The Greek version of the CIS-R was translated and back-translated using the procedure recommended by the World Health Organization http://www.who.int/substance_abuse/research_tools/translation/en/index.html. The psychometric properties of the Greek version of the CIS-R including its factor structure and internal consistency have been reported by Skapinakis et al. 2010 [46]. An internal consistency reliability analysis showed that item-test correlations ranged from 0.42 to 0.74, item-rest correlations ranged from 0.30 to 0.67 and Cronbach's alpha ranged from 0.84 to 0.87 with an overall alpha for CIS-R of 0.86. A test-retest reliability of the CIS-R was carried out in a subset of the present data set (two schools of the city of Ioannina with an interval between assessments of 2 weeks) and was found to be 0.84 [35].

For the purposes of the present study psychiatric morbidity can be assessed either in a dimensional way, using the total score on the CIS-R (by adding-up all 14 symptom dimensions), or in a categorical form using diagnostic categories. We chose to use the total score in our analyses because in that way we are able to adjust for the full spectrum of psychiatric morbidity including sub-threshold forms of illness. To better illustrate the association of bullying with the severity of psychiatric morbidity we defined four groups of severity: "no/minimal symptoms" (CIS-R score = 0-5), "subthreshold symptoms" (CIS-R score = 6-11), "mild symptoms" (CIS-R score = 12-17) and "severe symptoms" (CIS-R score > = 18). A score of 12 or above on the CIS-R indicates caseness [42,47], a score of 6-11 indicates some symptoms of mental disorder, a score of 0-5 indicates little evidence of mental disorder [47], while a score of 18 or more has been used as an indicator of severe morbidity [48].

### Assessment of school bulling

Involvement in bullying either as a perpetrator (bully others) or as a victim (being bullied by others) was investigated in the second phase of the study. We used two questions, one for being bullied and one for bullying others, taken from the revised Olweus Bully/Victim Questionnaire [49] which was also used in a WHO youth health study [50]. An introductory sentence defined bullying as follows:

"The next questions are about bullying. We say a pupil is being bullied when another pupil, or a group of pupils, says or does nasty and unpleasant things to him or her. It is also bullying when a pupil is teased repeatedly in a way he or she doesn't like. But it is not bullying when two pupils of about the same strength quarrel or fight."

The respondents were further asked how frequently they had been bullied or had bullied others during the last 2 months in school. The possible answers were: "many times a week", "about once a week", "2 or 3 times a month", "about once a month" and "not at all". Based on these responses we classified participants into the following groups: a) Not involved in bullying related behaviours, either as victims or perpetrators; b) being a victim but not a perpetrator; c) being a perpetrator but not a victim; d) being both a victim and perpetrator. Regarding the cut-off for frequency, we defined two variables, one including all instances of bullying-related behaviours (i.e. at least once a month) and the other focusing on high frequency only (i.e. at least once a week).

### Socioeconomic variables

We have used the following socioeconomic variables:

#### a) Parental Education

We assessed the educational status of both parents separately, since evidence shows that maternal socioeconomic characteristics have an equally, or even more, significant impact on child and adolescent health [51]. Pupils were asked to report their parents' highest educational level attained.

#### b) Parental Employment

Regarding employment, we chose to use the variable "employment type", which discriminates/distinguishes not only between employment status (employed--unemployed), but also between the different sectors of employment (self-employed, private or public sector employees). More specifically "employment type" was divided into six groups: "public sector employee"; "private sector employee"; "self-employed"; "pensioner"; "unemployed"; and "housewife" (as regards the mother's employment type) or "inability to work" (as regards the father's employment type). A residual category was also allowed ("else"). In Greece the legislative framework relating to the combination of family life and career (i.e. maternity leave, leave for breast-feeding/child bearing/illness, child/family allowances etc.) differs significantly between public and private sector [52]. Greek social research employs this kind of typology which links to the institutions and the way public life is organized in Greece. As a result the distinction between self-employed, private and public sector employees appears as very important for the country, since it determines in a great extent the way families live and function. Moreover, it is expected that the information lost by failing to ask the exact occupation, could be substituted by the information carried by the variable of education [53].

#### c) Financial status

We asked the adolescents to express their subjective view on any financial difficulties their family might experience recently. The specific question asked was: "How do you think that your family is doing financially?" The possible answers included: "My family experiences no financial difficulties", "My family experiences very few financial difficulties", "My family experiences some financial difficulties" and "My family experiences a lot of financial difficulties". This type of question takes into account adolescents' subjective view of their family's economic position.

#### d) School performance

In Greece, where typical 16-18 years-old adolescents have not yet entered the labour market, neither have they completed their education, own educational level or occupation cannot be used as a measure of personal social position. Academic performance in school has been often used in the literature as a measure of the position of the pupils in school [36,54,55]. We therefore included academic performance in our socioeconomic indicators by asking the participants to rate their school performance (based on their recent marks) in a scale with four choices ("excellent"; "very good"; "good"; "fair").

### Sociodemographic and other variables

In the first phase of the study information was selected from the students about several sociodemographic variables (grade the pupil is currently attending, gender, parent's age, parent's marital status, number of brothers and sisters). We also obtained adolescents' self-reports on their current body weight and height, we calculated the body mass index (BMI: kg/m^2^) for each student and we included this in our analyses as a categorical variable (BMI < 19, 19-22, 22-25, 25-30, > = 30) in order to be able to highlight any potential non-linear associations.

### Statistical analyses

The analyses were all conducted using the statistical software package STATA 10.0 (StataCorp, College Station, Texas). To take into account the potential effect of clustering of our data (since adolescents were nested into 25 schools) we first carried out a two-level logistic model (level 1: individuals, level 2: schools) in Stata using the gllamm command [56]. We also performed the models with the survey commands of Stata using school as the stratum. Results were very similar with both models and therefore in the paper we present the results using the survey commands because their use is more widespread in the literature. It should be noted that the effect of schools was negligible with an intraclass correlation coefficient close to zero (< 0.08). In all analyses we have used probability weights to take into account the stratified random sampling procedure.

The associations between involvement in bullying-related behaviours and psychiatric morbidity, socioeconomic and other variables were investigated using multinomial logistic regression models in Stata ("mlogit" command using the "svy" prefix" to obtain robust standard errors). The dependent variable was involvement in bullying (all frequencies, i.e. at least once per month) and the reference group was "not involved in bullying-related behaviours". We also present the analysis of the high frequency involvement only (i.e. at least once weekly) for comparison reasons and to check the sensitivity of our results in the change of the frequency cut-off. However, in the latter analysis, due to the small number of pupils with bullying-related behaviours in the fourth group of victims-perpetrators, odds ratios could not be calculated, therefore we only present odds ratios for victims vs. not involved and perpetrators vs. not involved.

Finally, since we have used categorical variables in our study, we have additionally calculated adjusted Wald tests in order to test the overall significance of our variables in the regression models, using the post-estimation command "test" in Stata.

## Results

### Description of the sample

Overall 5,614 students took part in the first phase of the study (55% girls, 41% 10th grade, 31% 11th grade, 28% 12th grade), while 2,431 students were interviewed in the second phase (59% girls, 39% 10th grade, 32% 11th grade, 29% 12th grade). A detailed table of the sociodemographic characteristics of the whole sample in both phases of the study is given in Additional file 1: Table S1. Due to the stratified sampling procedure there were more female than male students in the second phase.

### Prevalence of bullying behaviours

Tables 1 and 2 present the prevalence of bullying-related behaviours by gender. Table 1 presents the prevalence for all frequencies (at least once monthly) while Table 2 for higher frequency only (at least once weekly). Regarding all frequencies, it can be seen that 26.4% of the pupils were involved either as victims, perpetrators or both. The prevalence of being a perpetrator was approximately twice that of being a victim. Out of the 676 pupils who were involved in bulling-related behaviours, 136 were both victims and perpetrators (weighted percentage 18%). There were significant gender differences in the prevalence of bullying mainly because boys were more likely to be perpetrators compared to girls. Regarding frequent bullying, 4.1% of the pupils were involved and the same gender difference was also noted: boys were more likely to report that they had bullied others compared to girls; in contrast, there was no gender difference in victimization.

**Table 1:** Prevalence of bullying-related behaviours (at least once monthly or more) in Greek adolescents 16-18 years old attending senior high schools (N = 2,427)

	N,% (95% CI)		
	**Male**	**Female**	**Total**

Not Involved	N = 622, **65.7% (61.6-69.6)**	N = 1,129, **81.8% (79.3-84.1)**	N = 1,751, **73.6% (71.2-76.0)**

Victims only	N = 72, **6.7% (4.9-9.2)**	N = 145, **7.4% (6.1-8.9)**	N = 217, **7.1% (5.9-8.5)**

Perpetrators only	N = 214, **21.5% (18.3-25.2)**	N = 109, **7.4% (5.8-9.4)**	N = 323, **14.6% (12.7-16.7)**

Victims & Perpetrators	N = 80, **6.1% (4.5-7.9)**	N = 56, **3.4% (2.4-4.7)**	N = 136, **4.7% (3.8-5.8)**

^1 ^Actual number of observations; percentages in comparison are weighted to take into account the stratified random sampling procedure. Chi-square test was performed to examine sex differences. Chi_(3)_^2 ^= 113.6, p < 0.001

**Table 2:** Prevalence of bullying-related behaviours (at least once weekly or more) in Greek adolescents 16-18 years old attending senior high schools (N = 2,427)

	Male	Female	Total
Not Involved	N = 917, **93.9% (91.7-95.5)**	N = 1,400, 9**8.0% (97.2-98.6)**	N = 2,317, **95.9% (94.8-96.8)**

Victims only	N = 18, **1.3% (0.7-2.5)**	N = 28, **1.3% (0.8-2.0)**	N = 46, **1.3% (0.9-1.9)**

Perpetrators only	N = 50, **4.6% (3.2-6.6)**	N = 14, **0.7% (0.4-1.3)**	N = 64, **2.7% (1.9-3.7)**

Victims & Perpetrators	N = 4, **0.2% (0-0.6)**	N = 0, **0% (NA)**	N = 4, **0.1% (0-0.3)**

^1 ^Actual number of observations; percentages in comparison are weighted to take into account the stratified random sampling procedure. Chi_(3)_^2 ^= 37.52, p < 0.001

### Association between bullying-related behaviours (all frequencies) and socioeconomic status variables

Odds ratios and 95% confidence intervals for the associations of bullying-related behaviours with the sociodemographic and socioeconomic variables are shown in Tables 3 and 4 for all frequencies (at least once monthly) and for the high frequency (at least once weekly) respectively. We present odds ratios for victims, perpetrators and victims-perpetrators for all frequencies. For the high frequency category we only present odds ratios for victims and perpetrators only due to the very small number of victims-perpetrators in this frequency.

**Table 3:** Associations of bullying-related behaviours (at least once monthly) with socioeconomic variables and psychiatric morbidity.

	(base = Not involved in bullying-related behaviours) OR (95% CI)		
	
	Victims	Perpetrators	Victims-Perpetrators
**Female Gender**	**0.56 (0.37-0.85)**	**0.22 (0.15-0.33)**	**0.33 (0.20-0.55)**

**Grade**			
10th	1.00	1.00	1.00
11th	0.80 (0.49-1.29)	1.39 (0.95-2.04)	1.37 (0.77-2.43)
12th	0.63 (0.39-1.00)	1.09 (0.71-1.70)	**0.49 (0.26-0.90)**

**Father's Educational Level**			
Primary	1.00	1.00	1.00
Secondary Basic	1.04 (0.52-2.06)	1.00 (0.52-1.93)	1.30 (0.57-2.96)
Secondary Complete	0.75 (0.43-1.32)	1.04 (0.56-1.93)	0.84 (0.40-1.79)
Technological degree	0.65 (0.30-1.42)	0.98 (0.48-2.01)	**0.34 (0.13-0.93)**
University degree	0.95 (0.50-1.81)	1.22 (0.59-2.50)	0.98 (0.45-2.15)

**Mother's Educational Level**			
Primary	1.00	1.00	1.00
Secondary Basic	0.94 (0.48-1.83)	1.34 (0.67-2.67)	1.95 (0.81-4.67)
Secondary Complete	0.73 (0.42-1.27)	1.14 (0.63-2.07)	1.10 (0.51-2.37)
Technological degree	0.48 (0.22-1.02)	0.52 (0.24-1.15)	1.66 (0.65-4.24)
University degree	0.70 (0.29-1.67)	0.94 (0.45-1.96)	1.58 (0.62-4.05)

**Financial Difficulties**			
No	1.00	1.00	1.00
Very few	1.38 (0.85-2.23)	1.18 (0.79-1.76)	1.14 (0.67-1.95)
Some/A lot	1.22 (0.71-2.11)	1.33 (0.77-2.30)	1.41 (0.72-2.76)

**School Performance**			
Excellent	2.05 (0.90-4.66)	0.53 (0.26-1.11)	1.35 (0.61-3.01)
Very good	1.41 (0.83-2.39)	**0.53 (0.33-0.86)**	0.86 (0.43-1.71)
Good	1.41 (0.85-2.36)	0.79 (0.52-1.21)	1.23 (0.68-2.21)
Fair	1.00	1.00	1.00

**Father's Employment Type**			
Public sector employee	1.00	1.00	1.00
Private sector employee	1.02 (0.58-1.78)	0.87 (0.51-1.49)	0.70 (0.38-1.27)
Self-employed	1.21 (0.76-1.91)	1.61 (0.99-2.63)	0.92 (0.51-1.68)
Retired	0.77 (0.31-1.92)	**0.32 (0.14-0.71)**	0.89 (0.22-3.60)
Unemployed/Other	2.28 (0.86-6.05)	**2.32 (1.19-4.51)**	0.73 (0.24-2.26)

**Mother's Employment Type**			
Employee (public or private)	1.00	1.00	1.00
Self-employed	1.41 (0.72-2.76)	1.08 (0.63-1.88)	2.01 (0.84-4.78)
Unemployed	0.63 (0.29-1.39)	0.87 (0.41-1.88)	1.39 (0.47-4.10)
Looks after house	0.67 (0.40-1.11)	1.00 (0.65-1.57)	**2.11 (1.20-3.69)**
Retired//Other	0.47 (0.21-1.06)	0.86 (0.39-1.91)	**2.53 (1.16-5.52)**

**BMI**			
< 19	1.00	1.00	1.00
19-22	0.80 (0.48-1.33)	1.41 (0.84-2.37)	0.89 (0.47-1.69)
22-25	0.85 (0.47-1.55)	1.32 (0.73-2.41)	1.05 (0.51-2.13)
25-30	1.28 (0.59-2.79)	1.10 (0.57-2.13)	2.00 (0.94-4.22)
> = 30	2.42 (0.49-11.98)	1.85 (0.57-5.99)	1.41 (0.33-6.01)

**Psychiatric Symptoms**			
No symptoms	1.00	1.00	1.00
Subthreshold symptoms	**2.15 (1.03-4.50)**	**1.65 (1.04-2.60)**	**4.52 (1.94-10.54)**
Mild symptoms	**3.06 (1.43-6.52)**	**1.92 (1.22-3.03)**	**3.77 (1.67-8.52)**
Severe symptoms	**6.32 (3.30-12.09)**	**1.75 (1.08-2.86)**	**7.57 (3.33-17.23)**

Adjusted odds ratios of bullying-related behaviours in adolescents 16-18 years old attending senior high schools in Greece (N = 2,427)

OR: Odds ratio

CI: Confidence Interval

**Table 4:** Associations of bullying-related behaviours (at least once weekly or more) with socioeconomic variables and psychiatric morbidity.

	(base = Not involved in bullying-related behaviours)* OR (95% CI)	
	
	Victims	Perpetrators
**Female Gender**	**0.89 (0.38-2.07)**	**0.08 (0.04-0.19)**

**Grade**		
10th	1.00	1.00
11th	0.55 (0.26-1.18)	1.25 (0.61-2.56)
12th	**0.34 (0.15-0.77)**	**0.25 (0.10-0.61)**

**Father's Educational Level**		
Primary	1.00	1.00
Secondary Basic	1.87 (0.68-5.10)	1.63 (0.51-5.19)
Secondary Complete	0.85 (0.30-2.38)	1.09 (0.34-3.47)
Technological degree	**0.21 (0.04-0.92)**	0.67 (0.19-2.31)
University degree	1.05 (0.32-3.41)	0.60 (0.19-1.88)

**Mother's Educational Level**		
Primary	1.00	1.00
Secondary Basic	0.59 (0.19-1.80)	0.65 (0.18-2.30)
Secondary Complete	0.75 (0.30-1.84)	**0.24 (0.07-0.77)**
Technological degree	0.59 (0.15-2.35)	0.53 (0.13-2.20)
University degree	0.74 (0.17-3.14)	0.50 (0.11-2.25)

**Financial Difficulties**		
No	1.00	1.00
Very few	0.91 (0.41-2.02)	2.08 (0.94-4.59)
Some/A lot	1.50 (0.58-3.86)	2.93 (0.97-8.81)

**School Performance**		
Excellent	1.79 (0.49-6.52)	0.86 (0.28-2.65)
Very good	0.66 (0.25-1.74)	**0.21 (0.08-0.55)**
Good	0.83 (0.34-2.00)	0.62 (0.30-1.30)
Fair	1.00	1.00

**Father's Employment Type**		
Public sector employee	1.00	1.00
Private sector employee	1.73 (0.64-4.70)	1.30 (0.44-3.83)
Self-employed	1.16 (0.47-2.85)	**4.62 (1.91-11.19)**
Retired	0.41 (0.05-3.40)	0.65 (0.13-3.26)
Unemployed/Other	2.06 (0.56-7.62)	1.62 (0.32-8.08)

**Mother's Employment Type**		
Employee (public or private)	1.00	1.00
Self-employed	0.50 (0.12-2.07)	0.69 (0.22-2.17)
Unemployed	0.21 (0.02-1.95)	1.53 (0.33-7.15)
Looks after house	1.15 (0.47-2.81)	0.63 (0.26-1.50)
Retired//Other	0.22 (0.02-2.45)	0.20 (0.04-1.11)

**BMI**		
< 19	1.00	1.00
19-22	0.77 (0.31-1.90)	**4.94 (1.75-13.94)**
22-25	1.02 (0.38-2.77)	**3.13 (1.06-9.28)**
25-30	0.62 (0.19-2.01)	**5.49 (1.48-20.38)**
> = 30	**8.86 (1.89-41.77)**	0.50 (0.05-5.68)

**Psychiatric Symptoms**		
No symptoms	1.00	1.00
Subthreshold symptoms	1.52 (0.46-5.07)	**3.00 (1.11-8.06)**
Mild symptoms	1.24 (0.34-4.55)	**3.39 (1.21-9.50)**
Severe symptoms	**4.67 (1.51-14.47)**	**4.54 (1.52-13.56)**

Adjusted odds ratios of bullying-related behaviours in adolescents 16-18 years old attending senior high schools in Greece (N = 2,427)

* Please note that due to the small number of pupils with bullying-related behaviours in the fourth group of victims-perpetrators, odds ratios could not be calculated, therefore we only present odds ratios for victims vs. not involved and perpetrators vs. not involved

OR: Odds ratio

CI: Confidence Interval

#### Associations with victims

Victimization was significantly associated with psychiatric morbidity and male gender. From the socioeconomic indicators studied none was significantly associated in the adjusted model.

#### Associations with perpetrators

Being a perpetrator was significantly associated with psychiatric morbidity and male gender. From the socioeconomic indicators studied a lower school performance (Adjusted Wald test: F_3,2400 _= 2.57, *p *= 0.05; odds ratio for linear term: 1.31, *p *= 0.008) and father's employment status (Adjusted Wald test: F_4,2400 _= 5.92, *p *= 0.0001) were associated with being a perpetrator. For employment this was mainly due to the unemployment of the father that was associated with an increased risk of being a perpetrator and retirement of the father that was associated with a lower risk.

#### Associations with victims-perpetrators (both behaviours occurring at the same time)

Male gender and psychiatric morbidity were significantly associated with an increased risk of showing both behaviours at the same time (victims and perpetrators). In addition, being a younger pupil (attending 10th grade) was also associated with an increased risk (Adjusted Wald test: F_2,2400 _= 5.47, *p *= 0.004)

Regarding the socioeconomic indicators, mother's employment status was associated with being a victim-perpetrator (Adjusted Wald test: F_4,2400 _= 2.54, *p *= 0.04). This was mainly due to a higher risk for those pupils whose mothers were looking after the house or were retired. There was a non-significant trend for father's educational level to be associated with victims-perpetrators (Adjusted Wald test: F_4,2400 _= 2.10, *p *= 0.07), mainly due to a lower risk of being a victim-perpetrator in the category "technological degree" of father's educational level.

### Associations with socioeconomic status variables in high frequency only

As mentioned in methods we also present results for involvement in bullying in a higher frequency (i.e. at least once weekly) to check the sensitivity of our results in the change of the frequency cut-off.

Regarding victims the following variables were found to be associated with an increased risk: being a younger pupil (Adjusted Wald test: F_2,2400 _= 3.42, *p *= 0.03), having higher psychiatric morbidity (Adjusted Wald test: F_3,2400 _= 3.45, *p *= 0.02) and having a higher BMI (Adjusted Wald test: F_4,2400 _= 3.20, *p *= 0.01), mainly due to an increased risk of the overweight pupils (BMI > = 30). None of the socioeconomic indicators were found to be associated with an increased risk.

Regarding perpetrators, male gender, younger pupils and a higher psychiatric morbidity were also associated with an increased risk. BMI was associated in a non-linear way with a lower risk in the two extremes (Adjusted Wald test: F_4,2400 _= 3.18, *p *= 0.01). From the socioeconomic indicators studied a lower school performance (Adjusted Waldt test: F_3,2400 _= 3.30, *p *= 0.02) and father's employment (Adjusted Wald test: F_4,2400 _= 5.34, *p *= 0.0003) were found to be associated with an increased risk as was the case in the "all frequencies" analysis. However, for father's employment this was mainly due to the self-employment category of father and not that of unemployed. It should be noted that a linear term for financial difficulties (i.e. using the variable as a continuous one) had an odds ratio of 1.67 (95% CI: 1.01-2.84, *p *= 0.047) which was marginally significant. However, using the variable in a categorical form this was not significant (Adjusted Wald test F_2,2400 _= 2.04, *p *= 0.13)

## Discussion

### Main findings

In a sample of late adolescents attending senior high schools in Greece 26.4% of the pupils were involved in bullying-related behaviours either as victims, perpetrators or both. The prevalence of being a perpetrator was approximately twice that of being a victim, while approximately 18% of the pupils who were involved in bullying showed both behaviours. Frequent bullying (at least once weekly) was less common. In our study we confirmed the strong association of psychiatric morbidity with all types of bullying-related behaviours. The association with socioeconomic status however seems to be more complex. In victims we did not find any evidence for an association with the socioeconomic variables studied. In contrast there was some evidence that perpetrators (pupils who bully others) are more likely to come from families with an unemployed father. On the other hand, father's retirement was associated with a lower risk of perpetration. In addition the personal social position of the pupil in the school, as reflected in their school performance, is more likely to be lower in perpetrators. Economic inactivity of the mother (either housekeeping or retirement) was associated with an increased likelihood of being both a victim and perpetrator. These results were generally confirmed when we changed the cut-off of bullying frequency (from once monthly to once weekly), although in that case there was an association of bullying others (perpetrators) with self-employment of the father rather than unemployment. Of note, financial difficulties in the family did not seem to be associated with bullying-related behaviours, although in the case of the high frequency bullying there was a marginal result concerning financial difficulties and the likelihood of being a perpetrator.

### Comparison with other studies

Our study indicates that bullying is an important problem for Greek youth. As mentioned above, there is considerable variability among countries in the prevalence of bullying, which has been partly attributed to sociodemographic and cultural differences and partly to methodological inconsistencies [16]. Our results are consistent with studies conducted in different countries, which used similar instruments and a similar recall period [17]. Differences in the prevalence of bullying could also be explained by the fact that bullying is more prevalent among younger pupils [17]. Several studies report age differences in pupils' understanding of the term and experience of bullying [57,58] and results may not be comparable between studies using different age samples.

A strong and graded association was reported between psychiatric morbidity and both bullying behaviours. Similar associations have been presented by previous studies [21,59]. The cross-sectional design of our study does not allow us to draw any conclusions on the direction of this association. It could be argued that both directions of causality are possible. As a result the debate remains as to whether bullying-related behaviours precede the onset of mental health problems or whether people with emotional problems tend to get more involved in such behaviours. Longitudinal studies have shown that a history of bullying-related victimization predicts the onset of emotional problems, while previous emotional problems are not significantly associated with future victimization [5].

Our study has pointed out some interesting associations between socioeconomic indicators and the likelihood of being a perpetrator. Parental employment type and adolescent's school performance showed associations which are independent of the presence of psychiatric morbidity. Studies investigating bullying behaviours have often controlled for mental health problems using simple self-reported questionnaires [17,26]. In our study we used a fully structured psychiatric interview assessing a broad range of common psychological symptoms. As a result there is strong evidence that the socioeconomic associations reported here are independent of any possible psychiatric problems including sub-threshold symptoms.

Parental unemployment, especially of fathers, has been negatively associated with various aspects of adolescents' health and well-being [60], which may explain the associations found in our sample. Another possible mechanism mediating this association might be parental support, since parental unemployment reduces perceived parental support [61], which has been associated with less involvement in bullying-related behaviours [62]. As regards the association with the self-employment of the father, which has arisen in the analysis of the high frequency of the variables, it can be argued that self-employment in Greece is characterized by long and flexible working-hours, flexible holidays and considerable temporal income-variations, which may cause significant insecurity in the family context and also reduce perceived parental support.

Poorer academic achievement in school, which could be considered as an indicator of school adjustment and adolescents' own position in the school setting, has been previously associated with an increased risk of being a perpetrator [17]. In our study, a lower school performance was associated with an increased risk of perpetration, while there was some evidence of a J-type association in the high frequency only, with both fair and excellent school performance being associated more strongly with frequent perpetration. It can be argued that adolescents, who think that their school performance belongs to the extreme ends of the range, may feel that they do not belong to the average group when compared to their peers, a perception which could be very stressful for the adolescent, leading perhaps to antisocial behaviours like bullying.

Parental education as an overall variable was not associated with bullying perpetration or victimization. However, some subgroups of the variable were associated with bullying-related behaviours. The category "technological degree" of father's educational level was associated with a lower risk of being a victim-perpetrator and being a frequent victim, while the category "secondary complete" of mother's educational level was associated with a lower risk of being a frequent perpetrator. Associations with parental education have been reported by previous studies that also used pupils' self-reports, as well as with studies that used parental reports [63].

Financial difficulties of the family have been often used in studies investigating socioeconomic health inequalities of children and adults [34,64] and several studies confirm a relationship between low parental socioeconomic status and being a victim or a perpetrator [16,24]. In our study we did not find evidence that financial difficulties may be associated with bullying-related behaviours when all frequencies of bullying are included. When we focused however in high frequency (perhaps pointing to a more severe range of the spectrum) there was a trend for more financial difficulties in the family to be associated with an increased risk of being a perpetrator. However, this was dependent on the analysis (there was a marginally significant association only when the variable was treated as a continuous variable) and therefore we are unsure whether financial difficulties may have a negative impact on pupil's behaviour.

Regarding sociodemographic factors we confirmed that boys, overweight, obese and younger pupils are more frequently involved in bullying behaviours either as victims or as perpetrators [17,26,38,39]. An interesting finding in our study was the nonlinear relationship between the BMI and being a victim of bullying behaviours. Severe obesity (BMI > 30) was found to be associated with victimization only in the high frequency analysis.

### Limitations of the study

When interpreting the above mentioned findings the cross-sectional nature of our study should be taken into account, since it does not allow us to make any causal inference about the association between bullying and the socioeconomic indicators studied. Furthermore, our sample did not include adolescents attending technical vocational schools but only those attending senior high schools (approximately 75% of the school-attending adolescents of this age). Parental employment status was based on adolescents' self-report, which may result in some misclassification. However, this kind of misclassification is expected to be random. Moreover, the question about parental employment status did not include information about the exact occupation and as a result an official "occupational status" classification was not possible.

In order to calculate the body mass index of the students we have used adolescents' self-reports of their current body weight and height. Research has shown that adolescents' self-reports of height, weight and BMI are on the average valid representations of their measured counterparts. However, even when they occur, systematic errors in self-reported height, weight and BMI are negatively associated with the corresponding measured dimension and the prevalence of overweight based on BMI from self-reported measures is expected to be systematically underestimated relative to measured values [65]. As a result, even if these errors influence the results, this will be towards the null value, i.e. against our hypotheses.

Moreover, in this study we have used the variable of self-reported school performance and not variables based on objective data, such as official school grades or national examinations, which are expected to be more accurate measures of pupils' school performance. However, our objective in the current study was not to distinguish between academically good and worse students or investigate possible effects on adolescents' academic achievements, but to use a measure, which could reflect adolescents' own perceptions about their current position in the field where they are mainly active. This was the reason why we chose to use a subjective measure reported by the adolescent rather than objective data. We would like to note though that perceived academic achievement has been often used also in studies about bullying in schools [17].

Finally, we have used a subjective socioeconomic variable, namely adolescents' self-reports on the financial difficulties of their families. It has been suggested that directly questioning adolescents about their family's income can be unreliable [54]. In the literature financial difficulties of the family have been often used as a socioeconomic indicator in studies investigating socioeconomic health inequalities in populations of children and adults [34,64]. These studies have shown that more subjective indicators (such as financial difficulties, financial strain etc.) may be equally or even more important compared to more objective indicators of socio-economic status [64].

## Conclusions

Bullying in school is a substantial problem for Greek youth. This study has shown that perpetration was independently associated with some dimensions of adolescents' socioeconomic status, while victimization showed no socioeconomic associations. Our findings indicate that lower socioeconomic status as expressed by typical and other measures is related to an increased risk of being a perpetrator. The current findings may add to our understanding of possible risk factors for bullying behaviours in adolescence. The identification of potential risk factors plays a decisive role in the design of effective anti-bullying programmes. Several countries have adopted anti-bullying measures in schools and some of them have been found to be effective at least in the short-term [66]. More has to be done, however, to maintain the results over longer periods. Greece has only recently started discussing the implementation of a national policy against bullying. International experience combined with relevant research findings could contribute significantly to the organisation of effective anti-bullying programmes in the school setting, the benefits of which are expected to appear not only in adolescence but also in later life [67].

## Competing interests

The authors declare that they have no competing interests.

## Authors' contributions

KM helped in data collection, contributed to the statistical analyses and drafted the manuscript. PS was responsible for the conception and design of the study, helped in data collection, contributed to the statistical analysis and helped in the writing of the paper and interpretation of the results. TG helped in data collection, in the writing of the paper and interpretation of the results. SB helped in data collection, in the statistical analysis and interpretation of the results. RA made critical comments and helped in the interpretation of the results. SS helped in data collection and made critical comments that helped in the interpretation of the results. VM helped in obtaining funding for the study, in the writing of the paper and interpretation of the results. All authors read and approved the final manuscript.

## Supplementary Material

Additional file 1 **Basic description of the sample in the two phases of the study, of victims and of perpetrators of bullying behaviours**. Click here for file
