# Supplementary material for: Bullying behaviour in schools, socioeconomic position and psychiatric morbidity: a cross-sectional study in late adolescents in Greece
Source: Child Adolesc Psychiatry Ment Health. 2012 Feb 12;6:8. doi: 10.1186/1753-2000-6-8 (PMC3298787; doi:10.1186/1753-2000-6-8)
Supplement: Additional file 1 — Basic description of the sample in the two phases of the study, of victims and of perpetrators of bullying behaviours. [file 1753-2000-6-8-S1.DOC]

**Additional File 1:**

**Basic description of the whole sample in the two phases of the study, of victims and of perpetrators of bullying behaviours**

Table A1: Sociodemographic characteristics of the whole sample in Phase 1 (n=5614) and Phase 2 (N=2431) of the study, of victims and of perpetrators of bullying behaviours.

| **Variable** | **Number (%) or Mean (SD)** | | | |
| --- | --- | --- | --- | --- |
|  | **Phase 1 (n=5614)** | **Phase 2 (N=2431)** | **Victims** | **Perpetrators** |
| **Gender**  Male  Female | 2530 (45%)  3084 (55%) | 989 (41%)  1442 (59%) | 22 (44%)  28 (56%) | 54 (79%)  14 (21%) |
| **Grade**  10^th^ grade  11^th^ grade  12^th^ grade | 2281 (41%)  1772 (31%)  1561 (28%) | 942 (39%)  778 (32%)  711 (29%) | 25 (50%)  12 (24%)  13 (22%) | 31 (45%)  25 (37%)  12 (18%) |
| **Parent’s Family Status**  Married  Other/ Missing | 5012 (89%)  602 (11%) | 2145 (88%)  286 (12%) | 44 (88%)  6 (12%) | 55 (81%)  13 (19%) |
| **Father’s Age** | 47.97 (5.14) | 47.8 (4.9) | 39.2 (17.6) | 42.5 (15.6) |
| **Mother’s Age** | 42.60 (4.7) | 42.7 (4.6) | 34.7 (15.6) | 37.5 (14.4) |
| **Number of brothers/sisters**  None  One  Two or more | 446 (8%)  3087 (55%)  2035 (37%) | 194 (8%)  1.407 (59.5%)  762 (32.5%) | 6 (12%)  26 (52%)  18 (36%) | 4 (6%)  38 (56%)  26 (38%) |
| **Father’s Employment**  Employed – Public Sector  Employed – Private Sector  Self-employed  Unemployed  Retired  Other/Missing | 1828 (33%)  1183 (22%)  1949 (35.5%)  42 (0.5%)  290 (5%)  224 (4%) | 796 (33%)  511 (21%)  839 (34.5%)  17 (0.5%)  123 (5%)  145 (6%) | 11 (22%) 15 (30%)  17 (34%)  1 (2%)  6 (12%)  0 | 15 (22%)  14 (21%)  33 (48%)  2 (3%)  4 (6%)  0 |
| **Mother’s Employment**  Employed – Public Sector  Employed – Private Sector  Self-employed  Looks after House  Unemployed  Other/Missing | 1679 (30%)  1056 (19%)  741 (13%)  1720 (31%)  234 (4%)  184 (3%) | 681 (28%)  446 (18.5%)  347 (14%)  748 (31%)  109 (4.5%)  100 (4%) | 9 (18%)  13 (26%)  6 (12%)  1 (2%)  19 (38%)  2 (4%) | 21 (31%)  14 (21%)  9 (13%)  4 (16%)  17 (25%)  3 (4%) |
| **Father’s Educational Status**  Primary  Secondary Basic  Secondary Complete  Technological degree  University degree | 791 (14%)  849 (15%)  1589 (29%)  738 (13%)  1562 (28%) | 382 (16%)  344 (14%)  733 (30%)  309 (13%)  663 (27%) | 11 (22%)  8 (16%)  17 (34)  2 (4%)  12 (24%) | 16 (23.5%)  14 (20.5%)  16 (23.5%)  6 (9%)  16 (23.5%) |
| **Mother’s Educational Status**  Primary  Secondary Basic  Secondary Complete  Technological degree  University degree | 743 (13%)  784 (14%)  2086 (37.5%)  584 (10.5%)  1385 (25%) | 338 (14%)  365 (15%)  915 (37.5%)  233 (9.5%)  580 (24%) | 11 (22%)  6 (12)  19 (38%)  4 (8%)  10 (20%) | 12 (18%)  13 (19%)  16 (23.5%)  11 (16%)  16 (23.5%) |
| **Financial Difficulties**  None  Very little  Some  A lot | 1776 (32%)  3028 (54%)  675 (12%)  118 (2%) | 708 (29%)  1317 (54.5%)  328 (13.5%)  66 (3%) | 12 (24%)  22 (44%)  13 (26%)  3 (6%) | 15 (22%)  34 (50%)  12 (18%)  7 (10%) |
| **School Performance**  Excellent  Very Good  Good  Fair | 510 (9%)  1898 (34%)  2135 (38%)  1047 (19%) | 209 (8.5%)  751 (31%)  932 (38.5%)  526 (22%) | 4 (8%)  12 (24%)  21 (42%)  13 (26%) | 8 (12%)  8 (12%)  27 (39%)  25 (37%) |

SD: Standard Deviation
